# Postsurgical pyoderma gangrenosum after mastectomy with a familial component

**DOI:** 10.1093/jscr/rjae667

**Published:** 2024-10-22

**Authors:** Christine Courtney Rogers, Jordyn Nepper, Kassandra E Holzem, Chandler S Cortina

**Affiliations:** Division of Surgical Oncology, Department of Surgery, Medical College of Wisconsin, Milwaukee, WI 53226, United States; Department of Dermatology, Medical College of Wisconsin, Milwaukee, WI 53226, United States; Department of Dermatology, Medical College of Wisconsin, Milwaukee, WI 53226, United States; Division of Surgical Oncology, Department of Surgery, Medical College of Wisconsin, Milwaukee, WI 53226, United States; Medical College of Wisconsin Cancer Center, Milwaukee, WI 53226, United States

**Keywords:** postsurgical pyoderma gangrenosum, immunotherapy, breast cancer

## Abstract

Postsurgical pyoderma gangrenosum (PSPG) is a rare, ulcerative skin condition that presents a diagnostic challenge due to its similar presentation to infectious etiologies in the postsurgical period—often leading to gratuitous and unnecessary surgery and antibiotic use. We report a 37-year-old female with breast cancer who received neoadjuvant chemotherapy and immunotherapy and underwent bilateral skin-sparing mastectomies who developed delayed bilateral mastectomy skin flap necrosis secondary to PSPG. This case had rare factors associated with the development of PSPG such as preoperative systemic therapy and a familial component. This case underscores the importance of early recognition of this rare disease and appropriate management of PSPG to prevent unnecessary interventions and ensure an optimal outcome.

## Introduction

Postsurgical pyoderma gangrenosum (PSPG) is a subtype of pyoderma gangrenosum (PG), a diagnostically challenging and uncommon ulcerative skin disease characterized by innate immune system dysregulation and is autoinflammatory in nature [1]. The clinical presentation of PSPG is typically a painful lesion with a rapidly progressive bulla or necrotic ulcer that appears undermined with violaceous or erythematous borders following trauma of the skin [2]. The multiple types of PG include the most common ulcerative, and others such as bullous, vegetative, pustular, peristomal, and superficial granulomatous types [2]. Various presentations and similar appearance to other diseases can contribute to a delayed diagnosis [2, 3]. Histologic findings are important to further support the correct diagnosis and prompt treatment. These findings can be variable and depend on the age of the lesion and location of the specimen; typically, there is marked neutrophilic infiltrate, abscess formation and neutrophilic pustules within the epidermis and dermis [2]. PG is a diagnosis of elimination and is diagnosed with the constellation of characteristic histology and progressive ulceration with negative bacterial, mycobacterial, and fungal cultures [2]. Treatment includes wound care and analgesia with minor disease requiring topical corticosteroids, tacrolimus, and potential intralesional corticosteroid injections [2]. Severe disease requires oral corticosteroids [2]. Some patients require additional therapy with cyclosporine, colchicine, dapsone, minocycline, thalidomide, or biologics such as infliximab [2].

Surgery is a well-known cause of pathergy in PG, and there have been multiple reports of PSPG following breast surgery [4]. Furthermore, these patients may be at greater risk for PSPG given their potential use of neoadjuvant chemotherapy with immunotherapy as cases of neutrophilic eruptions like PG have been documented in association with immune checkpoint inhibitors, such as pembrolizumab [5]. Additional risk may exist in patients with genetic predisposition to PG [2, 6]. Familial association of PG is documented in a minority of cases and causative mutations are not well delineated [2, 3, 6]. In this study, we present a 37-year-old female with a diagnosis of PSPG following neoadjuvant chemotherapy and immunotherapy who underwent bilateral skin-sparing mastectomies with a family history of PSPG.

## Case report

A 37-year-old female diagnosed with clinical stage 3A (cT3 N0 M0), grade 3, triple-negative, invasive ductal carcinoma of the right breast received neoadjuvant carboplatin, paclitaxel, and pembrolizumab per current guidelines [7, 8]. She had an excellent response with clinical and radiologic disease regression. She elected to undergo bilateral skin-sparing mastectomies, axillary nodal staging, with delayed breast reconstruction.

**Figure 1:**
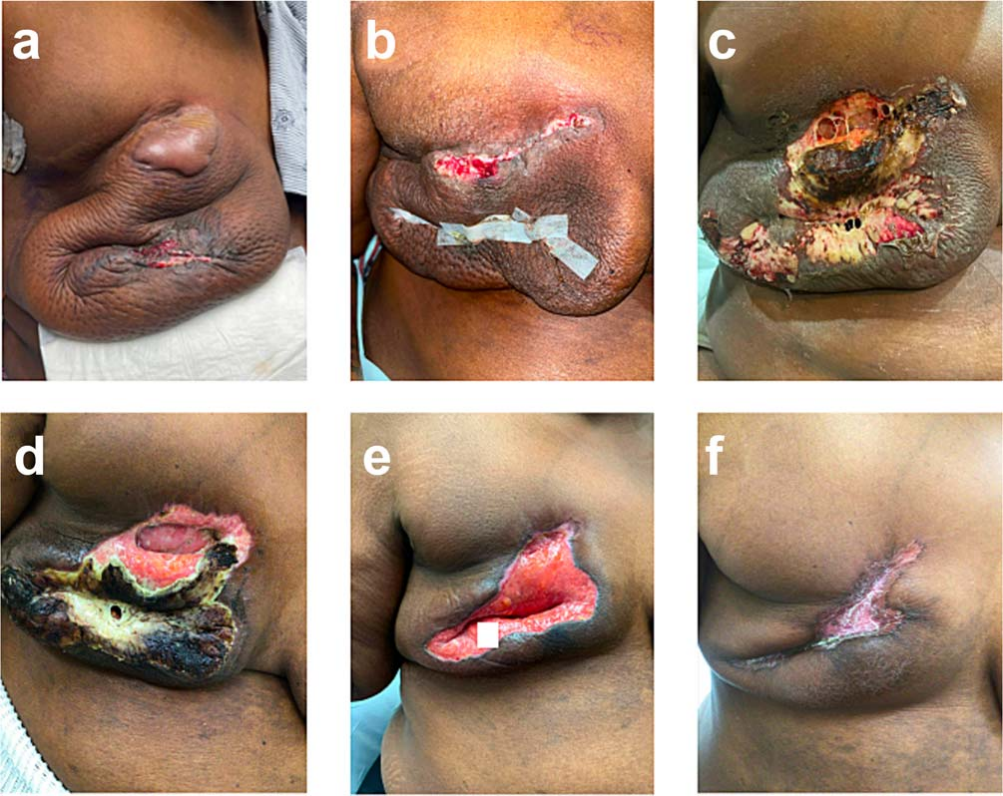
Right breast (a) POD 9, day three of antibiotics, large bulla, erythema, and incision-site drainage; (b) POD 12, two days following debridement and washout, day six of antibiotics; necrotic tissue removed, some dehiscence and erythema; (c) POD 15, started prednisone, significant dehiscence, induration, and large areas of necrosis; (d) POD 24, day nine of steroids, spread of necrosis is limited; (e) POD 40, day 25 of steroids, necrotic tissue removed; (f) POD 87, day 43 on infliximab, scar forming.

She experienced a normal postoperative course until postoperative day (POD) 6 when she became febrile and developed turbulent fluid in her surgical drains. She was empirically started on trimethoprim–sulfamethoxazole; drainage cultures grew pan-sensitive *Staphylococcus lugdunensis*. On POD 9, the patient was admitted with incisional changes (Fig. 1a). Concern for underlying infection prompted use of intravenous vancomycin, piperacillin, and tazobactam. Persistence of symptoms with minimal drain output raised concern for an undrained infectious fluid collection of her mastectomy beds prompting operative debridement of necrotic tissue and washout on POD 10 where full-thickness skin necrosis at the incision and beneath the skin bulla were seen. However, no purulent or undrained fluid was appreciated. Her skin was debrided to healthy tissue and closed primarily. Over the first 24 h, she did well; however, on POD 12, she became febrile with increased leukocytosis despite antibiotics, which were changed to piperacillin/tazobactam and linezolid. Repeat wound and blood cultures remained negative, but she developed progressive induration and wound breakdown (Fig. 1b). Given wound dehiscence, skin necrosis, and concern for PSPG, a punch biopsy was obtained.

Histopathological findings, with negative tissue cultures, confirmed the diagnosis of PSPG and antibiotics were stopped. She started on prednisone 40 mg/day, which increased to 80 mg/day (roughly 1 mg/kg dosing) after three days with improvement. This steroid dose was continued for 21 days and was then tapered over four weeks.

She was not a candidate for dapsone given persistent anemia following neoadjuvant chemotherapy and was treated with infliximab (started 5 weeks after completion of prednisone taper) as adjuvant pembrolizumab was planned per standard guidelines (Schmid, NCCN). Her wound improved with appropriate scarring (Fig. 1f). Notably, the patient’s daughter also had PSPG following a breast reduction years before—raising concern for a likely familial component to this patient’s case.

## Discussion

PSPG is a rare surgical complication that has frequent significant delays in diagnosis and can result in unnecessary surgical interventions and antibiotic use due to a broad differential diagnosis given the clinical picture of these patients [9]. As seen in this patient, PSPG is frequently misdiagnosed as a surgical site infection, skin necrosis, or wound dehiscence [9]. Once correctly diagnosed and patients receive correct therapy with immunosuppressants, recovery is often swift [2].

Familial association of PG is rare and estimated to occur in approximately 1.7% of cases [6]. Not only are there thought to be genetic predisposing factors leading to PG in families but also an association between PG and other immune related conditions such as inflammatory bowel disease, polyarthritis, and hematological disorders [6]. Additionally, this patient underwent neoadjuvant chemotherapy and immunotherapy prior to developing PSPG, which has been reported in some cases [10]. PG is associated with certain medications such as immune checkpoint inhibitors which activate antitumor T cell responses and can alter immune tolerance and are associated with various cutaneous effects, however, neutrophilic dermatosis such as PG is rare [5, 11]. This patient case illustrates a rare picture of PG with multiple infrequently or anecdotally associated factors with the development of PSPG. Further investigation is required to understand the underlying factors associated with the development of PG and clinicians must remain highly suspicious of PG in patients with this clinical picture.

Diagnosis of PSPG can be complex and requires a multidisciplinary team approach to diagnose and manage these patients to prevent gratuitous surgical intervention. Collaborations between surgeons, dermatologists, and pathologists are critical in cases where PG is suspected. Continued surgical site skin necrosis and infection despite surgical intervention and broad-spectrum antibiotics should prompt investigation for PSPG. PSPG should be considered in the differential diagnosis for patients with contributing factors such as familial predisposition, inciting tissue injury, and underlying malignancy treated with immunotherapy.
